# Matrices and Affinity Ligands for Antibody Purification and Corresponding Applications in Radiotherapy

**DOI:** 10.3390/biom12060821

**Published:** 2022-06-12

**Authors:** Aiying Xue, Saijun Fan

**Affiliations:** Tianjin Key Laboratory of Radiation Medicine and Molecular Nuclear Medicine, Institute of Radiation Medicine, Chinese Academy of Medical Sciences and Peking Union Medical College, Tianjin 300192, China

**Keywords:** matrices, particles, affinity ligand, antibody purification, radiotherapy, radiosensitizer, therapeutic radionuclide

## Abstract

Antibodies have become an important class of biological products in cancer treatments such as radiotherapy. The growing therapeutic applications have driven a demand for high-purity antibodies. Affinity chromatography with a high affinity and specificity has always been utilized to separate antibodies from complex mixtures. Quality chromatographic components (matrices and affinity ligands) have either been found or generated to increase the purity and yield of antibodies. More importantly, some matrices (mainly particles) and affinity ligands (including design protocols) for antibody purification can act as radiosensitizers or carriers for therapeutic radionuclides (or for radiosensitizers) either directly or indirectly to improve the therapeutic efficiency of radiotherapy. This paper provides a brief overview on the matrices and ligands used in affinity chromatography that are involved in antibody purification and emphasizes their applications in radiotherapy to enrich potential approaches for improving the efficacy of radiotherapy.

## 1. Introduction

Antibodies and their products have presented an approximately exponential growth in recent years [1] and have become the predominant class of new drugs for cancer treatment [2,3]. The therapeutic applications require high-purity antibodies [4], which are usually separated from complex mixtures containing various useless proteins. Antibodies can be isolated through a highly standardized platform, including pre-treatment, a capture step (protein A affinity chromatography), a polishing step (ion-exchange chromatography, hydrophobic interaction chromatography, etc.), and virus inactivation [5,6]. Chromatography has always been the mainstay of the antibody purification process, owing to its unparalleled scalability, robustness, and selectivity [7]. More importantly, all of the purification schemes for antibody production rely on the utilization of affinity chromatography to concentrate the product [5], thereby reducing the pressure of downstream purification.

Affinity chromatography employs different types of matrices and ligands. Protein A is the most commonly used affinity ligand for antibody purification and is covalently bonded to a natural or synthetic matrix [8]. However, protein A chromatography faces several disadvantages such as expensiveness, toxic ligand leakage [9], and a shorter lifetime of the resin [10], increasing the purification cost. To overcome these difficulties, a great deal of effort has been made to find or generate quality matrices and affinity ligands over the years.

Antibodies, especially monoclonal antibodies (mAbs), have been successfully utilized in combination with radiotherapy, providing an enhanced therapeutic effect in cancer treatment. Radiotherapy, harnessing high energy ionizing radiation to eradicate tumor cells, is a routine method for cancer treatment, and over half of all cancer patients need radiotherapy in current clinical therapy [11,12]. However, radiotherapy causes inevitable injury to surrounding healthy tissues and the emergence of radioresistance in cancer cells, which remarkably lowers the therapeutic efficiency or even leads to the failure of radiotherapy [13]. Hence, it has become an urgent priority to develop new regimens to enhance the therapeutic effects of radiotherapy.

Recent years have witnessed tremendous efforts in improving the efficiency of radiotherapy. Novel and effective radiosensitizers and carriers for therapeutic radionuclides (or for radiosensitizers) are two excellent representatives. Moreover, some radiosensitizers and carriers are similar to or the same as the matrices or affinity ligands for antibody purification. In other words, some matrices and affinity ligands (including design protocols) for antibody purification can either directly or indirectly (after further modification) play a vital role in improving the therapeutic efficiency of radiotherapy.

So far, there are few reports on the application of affinity chromatographic components in radiotherapy. This paper briefly reviews the chromatographic matrices and ligands for antibody affinity purification and highlights their applications in radiotherapy. At present, except for some published clinical experiences, these applications are still mainly in the experimental stage. This review will provide further available methods for increasing the efficacy of radiotherapy.

## 2. Chromatographic Matrices and Corresponding Applications in Radiotherapy

### 2.1. Chromatographic Matrices

**Figure 1:**
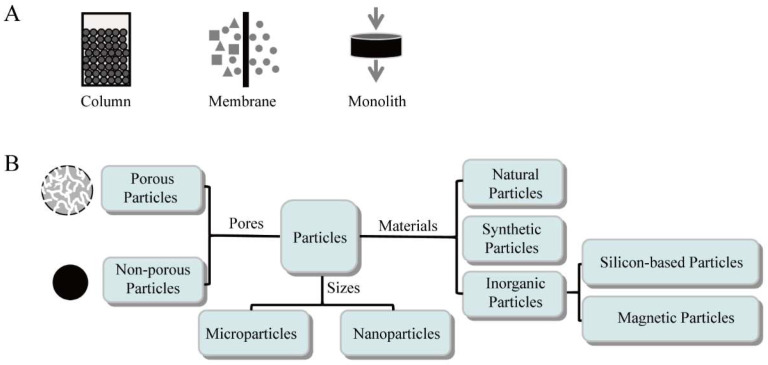
Chromatographic matrix for use in antibody affinity purification. (**A**) Formats of the stationary phase; (**B**) Classification of particles based on different criteria.

A prerequisite for affinity chromatography is a suitable matrix for the ligands. An ideal matrix should possess the characteristics of uniformity, stability, hydrophilicity, insolubility, minimum nonspecific absorption, and a large surface area for ligand attachment [14]. The stationary phase used for antibody affinity purification can be in the formats of packed-column, membrane, and monolith, as shown in Figure 1A, and the matrices packed in columns are adsorbents (mainly porous particles).

#### 2.1.1. Particles

The particles used as chromatographic matrices include microparticles and nanoparticles according to their sizes, and the micron-sized porous resins are the most commonly used matrices in packed-column chromatography. Based on the origin of the materials, particles are categorized into three groups [1], including natural, synthetic, and inorganic particles (Figure 1B).

Natural particles are usually prepared with agarose, cellulose, dextran, and their derivatives, and synthetic particles are commonly synthesized with polymethacrylate, polystyrene, and acrylamide derivatives [1,10]. For a detailed description of the natural and synthetic particles, please refer to other review articles [1,15].

The inorganic particles usually include porous silica particles and magnetic beads. Porous silica particles demonstrate a potential to be an alternative to traditional polymer supports on account of their easier regeneration, inexpensiveness, excellent flow properties, and easier surface modification [16,17]. Magnetic beads of different sizes are fabricated via entrapping magnetite within agarose, cellulose, polystyrene, or other polymeric materials, onto which ligands are fixed. Protein affinity separation using the magnetic beads offers the advantages of a low cost, robustness, rapid separation, few handling steps, and reduced system costs [4,18]. For example, an affinity sorbent was prepared by coupling protein A to magnetic monodisperse-porous SiO_2_ microspheres and employed to isolate immunoglobulin G (IgG) from rabbit serum in shorter isolation periods [19]. Nanometer-size magnetic beads are also applied in antibody purification. Without internal diffusion limitations, the non-porous structure of nanoparticles permits fast mass transfer of protein [4]. Cheng [20] prepared PEG-modified magnetic nanoparticles with a novel core-shell structure coupled with protein A to rapidly separate Omalizumab and IgG from cell culture supernatant and fetal calf serum, respectively.

Typically, affinity adsorbents have a porous structure to increase their surface area for protein adsorption, and these porous microspheres can be applied to columns. However, the pores are easily clogged with other proteins or foulants. On the contrary, non-porous microparticles or nanoparticles are not affected by particulate matter that presents in the mixture to be separated. Non-porous particles, especially magnetic beads, can be employed in antibody affinity separation in a non-column form. To date, the applications of magnetic separation have remained at a lab-scale.

#### 2.1.2. Membrane and Monolith

As an alternative to column chromatography, membrane chromatography that combines membrane filtration and liquid chromatography together has captured growing attention for antibody purification. Membranes offer the advantages of simplicity, ease of handling, a larger surface area, an improved mass-transfer efficiency, and an easier scale-up [21,22]. Affinity ligands, such as protein A/G [21], mimetic™ A2P [23], ligand 22/8 [24], and tryptamine [25], have been immobilized onto membranes for antibody purification from complex mixtures. For work regarding a detailed review of affinity membranes, please refer to the following article [22].

Monoliths are another alternative to packed columns, succeeding membrane adsorbers [7]. Monoliths are characterized by a single block of a homogenous stationary phase, a network of large, interconnected channels (or pores), endowing them with myriad advantages, including enhanced mass transfer, increased permeability, higher dynamic binding capacity, and lower preparation cost [26,27]. Monoliths have been extensively applied in antibody purification with different affinity ligands, such as protein A [28], L-histidine [29], and peptides [30,31]. Further discussion on affinity monoliths has been reported [26,32], and this review will not repeat it.

#### 2.1.3. Concluding Remarks

Despite the advantages of membranes and monoliths as affinity matrices, neither affinity membrane chromatography nor affinity monolith chromatography has replaced affinity column chromatography as the standard for antibody purification. The avoidance of regulatory issues is considered as the main reason [10]. Up to now, the most commonly used supports for affinity column chromatography are still the micron-sized porous resins (particles). Importantly, it is these particles that are associated with radiotherapy. Thus, in the following description of their applications in radiotherapy, the chromatographic matrices consist of these particles.

### 2.2. Applications of Particles in Radiotherapy

**Figure 2:**
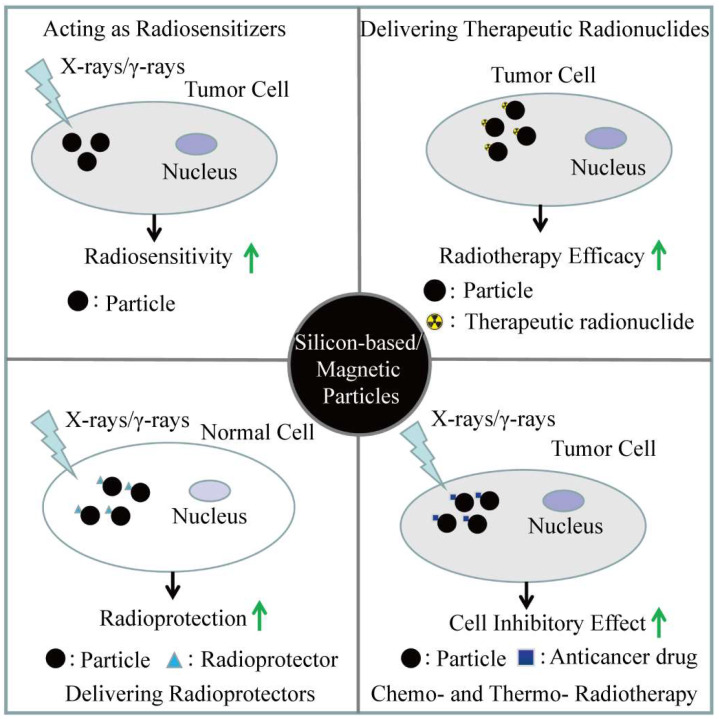
Applications of particles in radiotherapy. The particles mainly include silica-based and magnetic particles, and they can act as radiosensitizers, deliver therapeutic radionuclides and radioprotectors, and be applied in synergistic treatment.

Radiotherapy has been a routine method for clinical cancer therapy and is carried out with ionizing radiation that consists of particle radiation (α particles, β particles, etc.) or high-energy photon radiation (X-rays and γ rays) [33,34]. A higher dose of ionizing radiation is required in this therapy because of the low radiation absorption or the radioresistance of the tumors, causing severe damage to adjacent normal tissues. To enhance the efficacy of radiotherapy and decrease its radio-toxicity, novel and effective radiosensitizers or therapeutic radionuclide carriers have been developed. Some of the aforementioned microparticles and nanoparticles have been applied in radiotherapy either directly or with further modification. The applications mainly include acting as radiosensitizers, delivering therapeutic radionuclides, or radioprotectors [33,35,36], as shown in Figure 2.

#### 2.2.1. Acting as Radiosensitizers

Radioresistance has been a major reason for radiotherapy failure and subsequent tumor relapse [37] and the increasing radiosensitivity of tumor cells is thus greatly significant for attempt to enhance the efficacy and safety of radiotherapy. Radiosensitizers are promising agents to achieve this aim. To date, many highly effective and low-toxicity radiosensitizers have been developed to make various tumors more vulnerable to external radiation, and nanomaterials, such as silica-based and magnetic nanoparticles, are a type of effective radiosensitizers.

Silica-based nanoparticles have been used as radiosensitizers in radiotherapy. Silicon nanoparticles (<5 nm in size) clearly enhanced the reactive oxygen species (ROS) generation in rat glioma C6 cells upon exposure to X-rays, and the level of produced ROS was proportional to the received radiation dose, showing their ability to improve the efficacy of radiotherapy [38]. Aminosilanized oxidized silicon nanoparticles (NH_2_-SiNPs) could also significantly promote ROS production under X-ray irradiation in breast cancer and mouse fibroblast cells, and after reaching the mitochondria, NH_2_-SiNPs caused oxidative stress damage within the organelle [39], indicating that NH_2_-SiNPs has a potential role as a radiosensitizer for X-rays in tumor cells.

Magnetic nanoparticles can also act as physical radiosensitizers in radiotherapy [40,41]. They are highly biocompatible and have negligible toxicity to healthy tissues, and they can be directed and localized to tumors through an external magnetic force. Magnetic nanoparticles can produce cytotoxic effects on account of their ROS generation, leading to the damage of DNA and other cellular components [37,42]. Moreover, superparamagnetic iron oxide could enhance irradiation-induced DNA damage through catalyzing the generation of ROS [43], and this was confirmed on MCF-7 cells loaded with superparamagnetic nanoparticles [44]. Dextran-coated magnetic nanoparticles with increased chemical stability and biocompatibility [45,46] were also able to enhance the radiosensitivity of HeLa and MCF-7 cells, and the radiosensitivity increased with the dose rate or the concentration of nanoparticles [41].

In fact, there have been various kinds of nanoparticles that have displayed the ability to act as radiosensitizers. Nanoparticles based on heavy metal with a high atomic-number value, especially gold, have been typical radiosensitizers in recent years [34]. Likewise, these nanoparticles are beyond the scope of this review and are not discussed here.

#### 2.2.2. Delivering Therapeutic Radionuclides

Delivering therapeutic radionuclides into tumors is a promising strategy to enhance the effect of radiation on tumors and decrease radio-toxicity to neighboring normal tissues [33]. Generally, therapeutic radionuclides (such as ^131^I and ^90^Y [47]) are specifically delivered into tumors via suitable tumor-homing carriers, chiefly liposomes, microparticles, nanoparticles, micelles, dendrimers, and hydrogels [33,48]. Here, we describe the utilization of microparticles and nanoparticles in delivering therapeutic radionuclides.

Silica particles have been used to deliver therapeutic radionuclides. For instance, ^90^Y-labeled mesoporous silica particles have shown a high chemical durability even under weakly acidic conditions [49], and radiolabeled silica nanoparticles have presented an excellent stability in vivo [50]. Magnetic nanoparticles are another common type of particles used to deliver therapeutic radionuclides, such as the magnetic nanoparticles functionalized with PEG600 used as radioactive vectors to deliver ^90^Y [51]. Magnetic nanoparticles with other coatings, such as dextran, silica, human serum albumin, and phosphate ligands [33], have also been reported to deliver radionuclides. Radiolabeled magnetic nanoparticles could enhance tumor uptake and retention after intravenous administration [33]. In addition, there have been some commercially available radiolabeled microspheres, such as ^90^Y-labeled resin microspheres (SIR-Spheres).

These carriers are non-degradable, which not only leads to the inhibition of multiple administration, but also prohibits a precise therapeutic evaluation due to infeasibility of in vivo imaging [52,53]. Recently, biodegradable carriers have attracted increasing attention. Organic biomaterials, such as chitosan, are biocompatible, non-toxic, and biodegradable, and have been successfully used in the preparation of a new generation of radionuclide carriers [52]. For example, the biodegradable composite microspheres prepared from chitosan and collagen had a considerable biodegradability within 12 weeks. After being injected into rats with orthotopic hepatocellular carcinoma via the hepatic artery, the microspheres labeled with ^131^I effectively prolonged the median overall survival of the rats from 19 to 44 days [52].

In addition, several other biodegradable microspheres have already been developed to carry radionuclides, such as polylactic acid (PLA) microspheres loaded with ^186/188^Re and ^166^Ho and gelatin microspheres labeled with ^131^I [53]. These carriers are beyond the scope of this review and thus are not discussed.

#### 2.2.3. Delivering Radioprotectors to Healthy Tissues

The radioprotection of the surrounding healthy tissues is another promising approach to enhancing the efficacy and safety of radiotherapy. Most radioprotectors (radioprotective agents, which are basically free radical scavengers, antioxidants, or immunomodulators, which help to mitigate the radiation injuries) belong to organic molecular agents, which are insoluble in water and have a fast metabolism and thus a short circulation in the body, generating reduced radioprotective effects [54]. A drug delivery system is an ideal choice to compensate for these drawbacks. Nanoparticles are suitable as carriers for delivering radioprotectors into the body to increase the stability and circulation time of radioprotectors in vivo, eventually enhancing their bioavailability.

Inorganic nanoparticles are gradually used as carriers to assist radioprotectors in radioprotection [36,55]. For example, silica nanoparticles (20 nm) were used to load and deliver melanin, which was a naturally occurring pigment and possessed the properties of radioprotection. Melanin-coated nanoparticles minimized hematologic toxicity in irradiated mice, whereas they had no protective effect for metastatic melanoma tumors [55].

#### 2.2.4. Other Applications

Nanoparticles have also been utilized in the synergistic treatment combining radiotherapy and other treatments for cancer, such as chemo-radiotherapy and thermo-radiotherapy.

Chemo-radiotherapy, a combination of chemotherapy and radiotherapy [56], is a significant method for solid tumor treatment. Chemo-radiotherapy can increase the local efficacy of radiotherapy on primary tumors and may even inhibit the growth of distant metastatic tumors [57]. Although it improves tumor-killing, concurrent chemo-radiotherapy might have the risk of higher toxicities. Therefore, increasing the efficacy of chemo-radiotherapy and lowering its toxicity is of vital importance. Nanoparticles have been used to deliver chemo drugs for chemo-radiotherapy based on their unique characteristics, such as preferential accumulation in tumors and controlled drug release profiles [58,59]. Multifunctional mesoporous silica nanoparticles have been developed as vehicles to load an anticancer drug, selenocysteine (SeC) [60]. The SeC-loaded nanoparticles could significantly enhance the growth-inhibitory effect of cervical cancer cells induced by X-rays.

Thermo-radiotherapy is a combination of hyperthermia therapy and radiotherapy for cancer treatment. Hyperthermia treatment, carried out by locally raising the temperature of tumors, can inhibit the repair of irradiation-induced DNA breaks [61], make cancer cells in the G1 and G2/M phases more sensitive to radiotherapy [62], and increase tumor oxygenation [63], generating synergistic effects. Magnetic nanoparticles can be used in thermo-radiotherapy under an external alternating magnetic field to evenly heat the tumors [63,64]. For example, magnetic nanoparticles of 12 nm were directly injected into tumors of human glioblastoma multiforme patients, and subsequently heated under an alternating magnetic field. Furthermore, the treatment was conducted in combination with fractionated stereotactic radiotherapy, and finally presented a remarkable increase in overall survival to 13.4 months, compared with the control group treated with fractionated stereotactic radiotherapy alone (6.2 months) [65].

#### 2.2.5. Concluding Remarks

In radiotherapy, particles, nanoparticles in particular, can act as radiosensitizers, deliver radionuclides and radioprotectors, and apply to synergistic treatments, such as chemo-radiotherapy and thermo-radiotherapy. The characteristics of nanoparticles, such as their small size and high specific surface area, effective tissue penetration, and selective distribution to tissues and organs, are beneficial for increasing the efficacy of radiotherapy. Further progress is needed to promote the successful application of nanoparticles to clinical radiotherapy [66], such as developing nanoparticles with biosafety, improving tumor-specific accumulation and minimizing retention in vivo after radiotherapy, and effecting reproducible large-scale production under good manufacturing practice guidelines.

## 3. Affinity Ligands and Corresponding Applications in Radiotherapy

### 3.1. Affinity Ligands

Affinity ligands are the principal components of affinity chromatography. A suitable affinity ligand should possess these essential properties: a high affinity and specificity to a target protein, feasibility of immobilization, a retention of the binding capacity of the target protein after the attachment to the matrices, and possess stability under harsh washing and elution conditions [1,14]. Many ligands have been developed to purify antibodies, and these ligands are mainly classified into four broad categories, namely biospecific ligands, alternative scaffold proteins, synthetic ligands, and pseudobiospecific ligands. In addition, affinity tags, which are co-expressed as fusion partners with target proteins, are also able to act as affinity ligands for recombinant antibody capture [14]. The ligands are summarized in Table 1. In this review, we mainly focus on the ligands associated with radiotherapy and describe them in detail.

#### 3.1.1. Biospecific Ligands

Biospecific ligands refer to a group of naturally derived molecules, such as bacterially derived proteins, lectins, antigens, and nanobodies, which offer a high binding affinity and specificity to antibodies [67]. Bacterially derived proteins mainly include staphylococcal protein A, streptococcal protein G, and peptostreptococcal protein L, and are the most used affinity ligands for full-length antibodies. Lectins can specifically recognize and bind to the glycosylation sites on antibodies. Antigens can also act as affinity ligands to purify specific antibodies. Detailed description of these biospecific ligands can be found in other articles [1,68,69].

Nanobody, also known as V_H_H, is the recombinant, single-domain, and antigen-specific fragment of the heavy-chain-only antibodies that exist in the sera of camelids [70,71]. Nanobodies exhibit many peculiar characteristics, such as smaller size (15 kDa), high solubility and stability, refoldability, pH tolerance, and easy conjugation [71,72]. Moreover, their single-domain nature and strict monomeric behavior make it easy to produce them on a large scale by using microbial systems [1,14]. Nanobodies have been used to purify antibodies. For instance, a nanobody, separated from a naïve camelid single-domain phase display library, showed an affinity for the IgG-Fc fragment, and could bind to IgGs at a wide pH range (6.0–9.0) and NaCl concentrations. Under milder conditions, the bound IgGs could be efficiently eluted [72]. Nanobody resins have the potential to isolate IgGs from various complex samples [14], and have been commercialized, such as captureSelect^TM^ resins.

Despite their extensive use in antibody purification, biospecific ligands suffer from high cost, low stability, low binding capacities, ligand leakage, and limited life cycles [11,14]. To overcome these limitations, many different types of alternative ligands have been developed.

#### 3.1.2. Alternative Scaffold Proteins

Alternative scaffold proteins, a type of tailor-made protein, are generally built from smaller and structurally simpler frameworks. These frameworks usually contain altered amino acids or insertions, producing large variant libraries. Among these variants, specific ones with desired features can be isolated by selection techniques based on phage, ribosome, or bacterial or yeast display [68]. Several alternative scaffold proteins to act as affinity ligands for antibody purification have been developed, such as affibody, affitin, repebody, and monobody.

Affibody is developed from the B-domain of protein A, which antibodies bind to [73]. To enhance chemical stability, the B-domain is mutated early at key positions, and the obtained variant is denoted as the Z-domain, which represents the molecular origin of all affibodies [68,73]. The Z-domain retains its higher affinity for the Fc region of antibodies, while it almost completely loses its weaker affinity for the Fab region [68]. With the help of display techniques, Z-domain mutants are selected from vast combinatorial libraries, yielding many affibodies which exhibit specificity for antibodies of different classes [74,75]. For example, novel ligands for murine IgG1 were successfully acquired from a combinatorial ribosome display library of 10^11^ affibody molecules. One of the selected affibodies, termed Z_mab25_, showed high specificity for mouse IgG1 and was able to successfully capture mouse IgG1 from complex samples [75].

The other alternative scaffold proteins are not discussed in this review, and a detailed description can be found in other review articles [68,76].

#### 3.1.3. Synthetic Ligands

Generally, synthetic ligands are a type of low-molecular-weight compounds with properties of high chemical stability, robustness to sterilization, low cost, high versatility, and environmental friendliness [77]. Synthetic ligands mainly include three broad categories: peptidyl ligands, non-peptidyl ligands, and aptamers. In addition, synthetic polymer nanoparticles with a high affinity for antibodies are also included in this section.

##### Peptidyl Ligands

Peptidyl ligands refer to small peptides composed of a limited number of residues. They offer higher stability, cheapness, lower immunogenicity, and a moderate affinity for the target [68,78]. Peptidyl ligands include linear, cyclic, and dendrimeric peptides, based on their structures.

The linear peptidyl ligands of antibodies can be generated by synthetic solid-phase random peptide library screening. Typically, a family of linear hexapeptides are identified through this approach, which share a common sequence homology of histidine on the N-terminus followed by aromatic amino acid(s) and positively charged amino acids on the C-terminus. Three hexapeptides, HWRGWV, HYFKFD, and HFRRHL, can bind to IgG [79]. Particularly, HWRGWV possesses the ability to bind to different IgG subclasses from various species and has been successfully utilized in antibody purification [80,81,82]. Furthermore, HWRGWV was modified by replacing some residues with non-natural analogs to enhance its biochemical stability, while maintaining its target affinity and selectivity [83]. Moreover, linear peptidyl ligands of antibodies can also be efficiently developed by a biomimetic design strategy based on the structure of the receptor–ligand complex, which is commonly protein A-IgG. A group of octapeptide ligands were developed from a biomimetic design strategy that was based on the affinity motif of protein A in binding with human IgG and combined docking and molecular dynamics simulations [84]. A total of five octapeptides, FYWHCLDE, FYCHTIDE, FYCHWALE, FYCHNQDE, and FYCHWQDE, were identified as the affinity ligands of human IgG, and all of them were able to successfully capture high-purity and high-yield IgG from human serum [85,86].

Cyclic peptides possess attractive properties, such as a higher affinity and specificity towards targets, an increased resistance to enzymatic degradation, and the ability to act as the affinity ligands of antibodies [87]. Dendrimeric protein A mimetic (PAM), a typical dendrimeric peptidyl ligand, is a tripeptide tetramer and displays a broad specificity towards antibodies from different species [88]. Detailed descriptions of the cyclic and dendrimeric peptides can refer to reported reviews [68,89].

##### Non-Peptidyl Ligands

To overcome the weakness of peptidyl ligands that are susceptible to enzymatic degradation, non-peptidyl ligands have been developed either through the screening of chemical combinatorial libraries based on non-peptide backbones or through the rational design of small functional mimetics of natural Ig-binding proteins [14,68]. Commonly, non-peptidyl ligands show a high affinity and specificity, high durability, and a high binding capacity.

Ligands that mimic the binding mode of protein A or protein L and contain a triazine ring scaffold are the typical non-peptidyl synthetic ligands of antibodies. Artificial protein A (ApA) is the first fully synthetic non-peptidyl affinity ligand of IgGs based on triazine. It was generated by coupling Phe-Tyr, an essential dipeptide motif on protein A for IgG-protein A interaction, to a triazine scaffold [90,91]. Ligand 8/7 is another typical non-peptidyl affinity ligand, a synthetic mimic of protein L [92], and could selectively recognize and bind to both κ and λ light chains of IgG from different classes and sources [93].

##### Aptamers

Aptamers are short single-stranded nucleic acids (DNAs, RNAs, or combinations of these with non-natural nucleotides) that can adopt three-dimensional structures to bind to target molecules with high affinity and specificity [68,94]. Aptamers, with the properties of an increased stability, a mild elution, and a low cost, have been used as the affinity ligands of antibodies [68,94,95]. For example, Miyakawa et al. [95] developed a 23-nucleotide aptamer (Apt8-2) against a human IgG-Fc fragment, and Apt8-2 bound to the human IgG with high specificity and affinity. The interaction between Apt8-2 and IgG was stable under extreme conditions and vulnerable to neutral buffers, allowing for a gentle elution. Apt8-2-based affinity matrix supported IgG purification from human serum with a nearly equivalent purity and yield to protein A resin. Additionally, aptamers have the potential to monitor antibody production and control quality [96].

##### Polymer Nanoparticles

Polymer nanoparticles (NPs) can also act as the affinity ligands of antibodies [97,98,99]. For example, the polymer hydrogel NPs (50–65 nm) showed high affinity for IgG, while little affinity for other proteins [97]. Their binding domain on IgG overlapped with that of protein A. In addition, synthetic nanogel particles that bound to the IgG-Fc fragment could be immobilized on a matrix to reversibly capture IgG [98]. The inexpensive and stable polymer NPs with the capacity of selectively binding to antibodies are significant alternatives to natural protein ligands for applications in antibody affinity purification.

#### 3.1.4. Pseudobiospecific Ligands

Pseudobiospecific ligands are a type of alternative ligands that take advantage of the intrinsic properties of antibodies at the molecular level, and they are developed based on multiple non-covalent forces that involve the interaction of antibodies with affinity ligands [1,14]. The affinity of the pseudobiospecific ligands is relatively lower, but sufficient to ensure their specificity towards target antibodies [1]. Various pseudobiospecific ligands, such as hydrophobic [100], thiophillic [101], chelating metal ions, hydroxyapatite [102], and mixed mode ligands [1,14], have been developed and used in antibody purification. In addition, some amino acids, such as L-histidine [29] and L-tryptophan [103], are also able to capture antibodies. A detailed introduction can be found in previous reviews [1,14].

**Table 1:** Affinity ligands of antibody used in affinity chromatography and their main characteristics.

Category	Example (Ref)	Main Characteristic	
		Advantage	Disadvantage
Biospecific ligand	Bacterially derived protein [1,68,69]	Bacterially derived protein: Common ligands for full-length antibodies from various species	Biospecific ligand: 1. High cost; 2. Low binding capacities; 3. Ligand leakage; 4. Limited life cycles
	Staphylococcal protein A		
	Streptococcal protein G		
	Peptostreptococcal protein L		
	Lectin [1]	Specifically recognize and bind to the glycosylation sites on antibodies	
	Antigen [1]	For specific antibody purification	
	Nanobody [72]	Nanobody:	
		1. Single-domain nature;	
		2. Smaller size (~15 kDa);	
		3. High stability and solubility;	
		4. Refoldability and pH tolerance.	
Alternative scaffold protein	Affibody [74,75]	Affibody:	Alternative scaffold protein: Susceptibility to enzymatic degradation
		1. Tailor-made protein;	
		2. High chemical and thermal stability	
	Affitin [68]	Tailor-made protein	
	Repebody [68]		
	Monobody [68]		
Synthetic ligand	Peptidyl ligand [79,85,86,87,88]	Peptidyl ligand:	Peptidyl ligand: Susceptibility to enzymatic degradation
		1. Higher stability;	
		2. Lower immunogenicity and cheapness;	
		3. Gentle elution.	
	Non-peptidyl ligand [90,91,92,93]	Non-peptidyl ligand:	
		1. High affinity and specificity;	
		2. High durability and binding capacity.	
	Aptamer [94,95]	Aptamer:	
		1. Increased stability;	
		2. Mild elution;	
		3. Low cost.	
	Polymer nanoparticle [97,98,99]		
Pseudobiospecific ligand	Hydrophobic ligand [100]	Pseudobiospecific ligand: 1. Affinity: relatively lower but sufficient to ensure their specificity and selectivity towards target antibodies; 2. Affinity: relatively lower but sufficient to ensure their specificity and selectivity towards target antibodies;	Utilization commonly in combination with other antibody purification methods
	Thiophillic ligand [101]		
	Chelating metal ions		
	Mixed mode ligand [1,14]		
	Single amino acid		
	L-histidine [29]		
	L-tryptophan [103]		
Affinity tag	His_6_-tag [14]	For recombinant antibody purification	

#### 3.1.5. Concluding Remarks

To date, protein A is still one of the most commonly used affinity ligands in antibody production. New engineered protein A variants with excellent properties, such as higher binding capacity, stronger alkaline tolerance, and a milder elution condition, have been developed [69]. On the other hand, many alternative ligands have also been developed. Pseudobiospecific ligands are expected to be cost-effective and robust alternatives, but they are often used in combination with other purification methods. Developments in combinatorial libraries, in vitro selection techniques, and protein engineering have promoted the emergence of alternative scaffold proteins, while these proteins are susceptible to enzymatic degradation. The rational designing and high-throughput screening of ligands have facilitated the generation of synthetic ligands, which have presented substantial growth [68]. Synthetic ligands, mainly including peptides, non-peptidyl ligands, and aptamers that represent cheapness, scalability, and stability, are highly desirable.

### 3.2. Applications of Affinity Molecules in Targeted Radiotherapy

**Figure 3:**
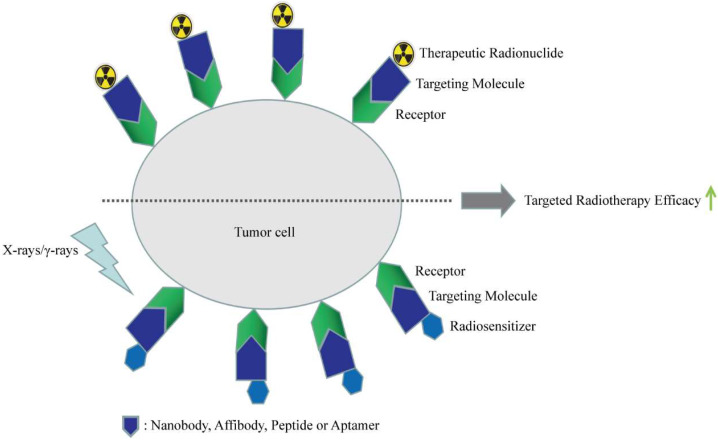
Applications of affinity molecules in radiotherapy. The affinity molecules mainly include nanobody, affibody, peptide, and aptamer, and they can carry therapeutic radionuclides and radiosensitizers to enhance the efficacy of radiotherapy.

High affinity and specificity towards targets are two of the most prominent characteristics of affinity ligands. The two properties are the pivotal requirements of ligands for targeted radiotherapy, a strategy to address the non-selectivity of radiation, thereby improving the selectivity of radiotherapy and its minimizing side effects [104]. Some of the aforementioned affinity ligands, such as antibody fragments, alternative scaffold proteins, peptides, and aptamers, can also be used as targeting moieties for ligand-based targeted radiotherapy. These molecules can either directly target tumors or be conjugated with suitable carriers to act as targeting moieties. Therapeutic radionuclides or radiosensitizers can be carried by the targeted delivery systems to enhance radiotherapy efficacy, as shown in Figure 3. In this section, different affinity molecules used in targeted radiotherapy are discussed, and their classification criteria keep consistent with those in Section 3.1.

#### 3.2.1. Biospecific Molecules: Mainly Nanobody

The biospecific molecules used as targeting moieties for ligand-based targeted radiotherapy mainly involve antibodies and antibody derivatives. They can target specific markers on cancer cells to deliver either therapeutic radionuclides [105] or radiosensitizers [106,107,108], and a detailed description can be found in the following articles [106,109].

Nanobodies, the smallest functional antigen-specific fragments from the heavy-chain-only antibodies [110], have been used as targeting carriers to specifically deliver therapeutic radionuclides to cancer cells. As targeting agents, nanobodies offer high stability and solubility, rapid blood clearance and low immunogenicity, particular suitability for penetrating tumor tissue, and an excellent specificity against all possible targets due to their ability to detect the hidden and inaccessible epitopes of target antigens [105,110]. Nanobodies labeled with therapeutic radionuclides have been investigated in preclinical models. For instance, ^177^Lu-labeled anti-human epidermal growth factor receptor 2 (HER2) nanobody was demonstrated to efficiently target HER2^pos^ xenografts, all while maintaining a low level of radioactivity in normal organs. The treatment of mice with small HER2^pos^ tumors by weekly intravenous injections of ^177^Lu-labeled anti-HER2 nanobody could lead to an almost complete blockade of tumor growth. On the contrary, tumors grew exponentially in untreated mice or in mice treated with a non-targeting nanobody [111]. Many other radiolabeled nanobodies, such as ^177^Lu-labeled anti-epidermal growth factor receptor nanobody [112], ^89^Zr-labeled anti-hepatocyte growth factor nanobody [113], and ^211^At-labeled [114] or ^225^Ac-labeled [115] anti-HER2 nanobodies, could enhance targeting in vivo and could potentially be used as targeting vehicles in targeted radionuclide therapy (TRNT).

In addition, radiolabeled nanobodies have also been applied in diagnostics for noninvasive molecular imaging to determine the biodistribution of radiopharmaceuticals in the body [109,110,116]. Using them for both diagnosis and therapy may be a promising strategy for guiding TRNT towards a successful outcome [109,116].

#### 3.2.2. Alternative Scaffold Proteins: Mainly Affibody

Among the above alternative scaffold proteins, affibody molecules have been applied in targeted radiotherapy. Their small size endows them with the advantages of effective tissue penetration and an ease of chemical synthesis. They are capable of being used as ‘naked’ proteins or as conjugates to deliver therapeutic radionuclides or radiosensitizers [105], and the affibody Z_HER2_ with a high affinity for HER2 receptors is the most widely used affibody in targeted radiotherapy.

Affibody molecules labeled with therapeutic radionuclides have been applied in targeted radiotherapy. For example, ^125^I-labeled affibody (Z_HER2:4_) could be internalized specifically in HER2 overexpressing cells [117]. However, the application of radiolabeled affibody to TRNT is prevented by a high renal reabsorption [105]. To overcome this limitation, one attempt was to fuse an affibody with an albumin-binding domain (ABD) [73,105,118]. For instance, the dimeric affibody molecule (Z_HER2:342_)_2_ was fused with ABD and labeled with ^177^Lu. The radiolabeled conjugate could bind specifically to HER2-expressing cells and tumors and enabled a 25-fold reduction of renal uptake, completely preventing tumor formation [118].

Affibody molecules can also deliver radiosensitizers in targeted radiotherapy. For example, Z_HER2:342_ molecules were coupled to gold nanoparticles, an X-ray radiosensitizer, and the conjugate could improve the ablation effect of X-ray radiation on HER2-overexpressing cancer cells [119]. Inorganic nanoparticles as radiosensitizers were also successfully delivered by a Z_HER2_-modified carrier to a target tumor, exhibiting an antitumor effect in combination with X-ray irradiation [120].

#### 3.2.3. Synthetic Ligands: Peptidyl Ligands and Aptamers

##### Peptidyl Ligands

Peptidyl ligands, which demonstrate the abilities of easily penetrating tissues, a rapid clearance from the blood, and a low antigenicity, have been used as targeting vectors for therapeutic radionuclides or radiosensitizers in targeted radiotherapy [121]. Their selectivity primarily depends on non-immunogenic mechanisms like receptor–ligand binding [122].

Radiolabeled peptides that can bind to receptors on tumor cells with high specificity and affinity hold great potential for targeted radiotherapy [121]. For example, ^90^Y labeled Arg-Gly-Asp (RGD) peptides, the most common peptide used for targeting, were used to target the α_v_β_3_ integrin on the neovasculature of OVCAR-3 ovarian carcinoma xenografts and could delay tumor growth [123]. ^90^Y- and ^177^Lu-labeled *E.coli* heat-stable enterotoxin analogs could specifically target the guanylate cyclase C receptor that was highly expressed on the surface of human colorectal cancer cells, and were applied in peptide receptor radiotherapy [124]. On the other hand, radiosensitizers, such as nanoparticles, have also been carried by peptidyl ligands to decrease cell viability and inhibit the invasive activity of cancer cells [104], enhancing the treatment efficacy of targeted radiotherapy.

##### Aptamers

Aptamers are DNA or RNA sequences with a secondary structure endowing them with the capacity for binding to target molecules with high affinity and specificity [94,125]. Aptamers offer many advantages, such as a small molecular weight, lack of immunogenicity, an ease of chemical synthesis, and superior tissue penetration [125]. Aptamers have a potential to act as targeting ligands for cell surface receptors, and they have been applied in targeted radiotherapy.

Aptamers could deliver therapeutic radionuclides to enhance antitumor effects. A DNA aptamer U2, targeting U87 cells overexpressing epidermal growth factor receptor variant III (EGFRvIII), could enhance the radiosensitivity of U87-EGFRvIII cells in vitro, and ^188^Re-labeled U2 was able to effectively inhibit the growth of U87-EGFRvIII xenografts in nude mice [126]. Aptamers are also excellent potential candidates for the targeted delivery of radiosensitizers, such as nanoparticles, to tumor antigens on the surface of cancer cells. For example, As1411 is a guanine-rich DNA aptamer with high specificity and affinity to nucleolin receptors which are only overexpressed on the plasma membrane of cancer cells. The specific interaction between As1411 and nucleolin has been used to mediate the highly specific targeting of radiosensitizers to cancer cells [126,127,128,129]. As1411-conjugated gold nanoparticles [128], gold nanoclusters [126], or silver nanoparticles [127,129] have been proved to be capable of acting as efficient radiosensitizers for cancer targeting treatment.

#### 3.2.4. Concluding Remarks

Targeting molecules are vital components in ligand-based targeted radiotherapy, and their selection is mainly determined by receptors on the surfaces of target cells [126]. Nanobodies, affibodies, peptides, and aptamers can carry therapeutic radionuclides and radiosensitizers to effectively inhibit or even eradicate tumors and minimize side effects. These affinity molecules of relatively small size show prominent advantages in radiotherapy, such as low immunogenicity, superior tissue penetration, and rapid blood clearance. Moreover, affibodies, peptides and aptamers are easy to chemically synthesize, allowing stable and repeatable production.

## 4. Discussion and Conclusions

In the past few decades, affinity chromatography has been the main technique employed for antibody purification, and protein A chromatography has been the predominant standard method [14]. Nonetheless, this method could not provide a solution to the stability and cost issues related to antibody purification, resulting in the need for further research to develop improved or alternative approaches to isolating antibodies.

Extensive research has been done to develop more advanced or novel matrices for antibody affinity chromatography. Microparticles are the most commonly used resins in packed-column chromatography. The optimization of pore size, structure, and volume has enhanced the mass transfer and dynamic binding capacity of antibodies in the microparticles [7]. An extended lifetime and an improved stability towards the strong cleaning and sanitizing chemicals of resins has been achieved. Moreover, the non-porous structure of nanometer-size particles permits a faster mass transfer of protein. Nanoparticles, especially magnetic nanoparticles, have been employed in antibody separation in a non-column form. Affinity magnetic separation can help alleviate concerns over speed, production, and affordability in antibody purification [4]. In addition, chromatographic membrane and monolith are promising alternative formats to the packed columns applied in antibody affinity purification.

Great efforts have also been made to improve the performance of protein A or develop novel affinity ligands for antibody affinity chromatography. Improved engineered protein A variants with excellent properties, such as higher binding capacity and stability, have been used in commercial resins. With the advancement of rational design methods and the screening technology of combination libraries, novel and effective affinity ligands with desired properties have been successfully developed for application to antibody purification [77]. These ligands mainly contain alternative scaffold proteins, peptides, non-peptidyl ligands, and aptamers. Among them, the peptides binding to the Fc-region of antibodies are attractive ligands for antibody purification. Linear short peptides have been widely studied, and cyclic peptides are also considered as feasible options due to their enhanced specificity, conformation rigidity, and superior enzymatic stability in comparison with linear peptides [89]. In addition, aptamers with unique features like high chemical stability, high shelf life, and low immunogenicity have also been explored as non-proteinaceous affinity ligands of antibodies.

Among the chromatographic components used for antibody affinity chromatography, some matrices and affinity ligands can also play a vital role in improving the therapeutic efficiency of radiotherapy. Microparticles and nanoparticles can act as radiosensitizers, deliver radionuclides and radioprotectors, and synergize with other treatments for cancer. In particular, nanoparticles (<50 nm in size) are able to easily enter most cells and (<20 nm in size) pass through blood vessel endothelium [130], which is beneficial to increasing the efficacy of radiotherapy. Regarding affinity molecules, nanobodies and molecules of non-antibody fragments can enhance the selectivity of radiotherapy and minimize its side effects. These molecules can deliver therapeutic radionuclides or radiosensitizers to target tumors. In particular, the affinity molecules of relatively small size, like affibodies, peptides, and aptamers, are attractive targeting moieties for ligand-based targeted radiotherapy, due to their ease of chemical synthesis, low immunogenicity, rapid blood clearance, and superior tissue penetration.

Although some chromatographic components can be used in radiotherapy, they might not be completely suitable for direct usage in radiotherapy, on account of their different intended applications determining the corresponding properties. When nanoparticles are used in radiotherapy, their biosafety is the vital issue. At present, most nanoparticles are undegradable, and their long-term accumulation in the body would cause biosafety concerns [109]. Therefore, before clinical applications, nanoparticles must be systematically evaluated based on their biocompatibility, biodistribution, biodegradability, and clearance. Developing biodegradable nanoparticles with an improved tumor-specific accumulation is a promising strategy for improving the efficacy of radiotherapy and decreasing radio-toxicity.

Notably, affinity molecules that may be used for both antibody purification and as a part of radiotherapy differ in that immobilized ligands (used in chromatography) cannot be directly used in radiotherapy—only in their soluble form. Of course, their high specificity and affinity are not the only requirements. They also should withstand several tough regeneration procedures in affinity chromatography, while also possessing suitable in vivo kinetic parameters in radiotherapy [73,105]. Nevertheless, the size and chemical stability of affinity molecules, as well as the manufacturing cost, shelf life, and intellectual property restrictions, are important for applications of both affinity chromatography and radiotherapy. Moreover, the approaches to generating novel ligands, such as peptidyl ligands and aptamers, for antibody affinity purification are efficient for designing and screening new ligand molecules for targeted radiotherapy [108]. The overexpression of cancer cell surface receptors and surface bound antigens represents the molecular basis for the rational design of targeted radiotherapy, and some of the receptors or antigens may serve the individualized design of cancer treatment. The ligand molecules can be rationally designed by improving the performance of natural affinity ligands or by being discovered through combinatorial libraries [77]. Considerable efforts have been directed towards ligand development for targeted radiotherapy. For instance, some radiolabeled peptides have emerged as promising novel molecules for targeting cancers over the past few years, but only few of them could reached clinical trials [108]. The rapid development of big data and artificial intelligence will promote the discovery of novel ligands for targeted radiotherapy and may even improve the precision of radiation treatments.

Additionally, advances in radiation treatments made using new technologies, such as intensity-modulated radiotherapy, stereotactic body radiotherapy, and MRI-guided radiotherapy, have significantly improved the efficacy of radiotherapy and reduced its toxicities [131,132,133]. In particular, metabolic radiotherapy is a type of radiotherapy treatment that is carried out by introducing radioactive compounds into the body. The radioactive compounds are metabolized only at the target location to destroy the malignant cells without affecting healthy tissues [134,135]. Moreover, the applications of chromatographic matrices and affinity ligands in radiotherapy may be limited and do not represent a therapeutic opportunity for all cancers at present. However, this paper provides the first overview of the applications of affinity chromatographic components in radiotherapy, and can significantly enrich the potential available methods for improving the efficacy of radiotherapy and decreasing radio-toxicity.

## Data Availability

Not applicable.
